# Socioeconomic disparities in achieving a live birth after initiating ART treatment: a national register-based study among women in Denmark

**DOI:** 10.1093/hropen/hoag032

**Published:** 2026-04-09

**Authors:** Rikke B Uggerhøj, Andrea R Skaaning, Frederik N Kyhl, Lone Schmidt, Ditte Vassard

**Affiliations:** Section of Social Medicine, Department of Public Health, University of Copenhagen, Copenhagen, Denmark; Section of Social Medicine, Department of Public Health, University of Copenhagen, Copenhagen, Denmark; Fertility Clinic, Department of Gynaecology, Fertility and Obstetrics, Copenhagen University Hospital, Rigshospitalet, Copenhagen, Denmark; Section of Social Medicine, Department of Public Health, University of Copenhagen, Copenhagen, Denmark; Section of Social Medicine, Department of Public Health, University of Copenhagen, Copenhagen, Denmark; Fertility Clinic, Department of Gynaecology, Fertility and Obstetrics, Copenhagen University Hospital, Rigshospitalet, Copenhagen, Denmark

**Keywords:** assisted reproductive technology, socioeconomic factors, live birth, educational level, income, labour market attachment

## Abstract

**STUDY QUESTION:**

How are educational level, labour market attachment, and income associated with achieving a live birth following ART treatment initiation among women in Denmark?

**SUMMARY ANSWER:**

Higher educational level, labour market attachment, and higher income were strongly associated with achieving a live birth after initiating ART treatment among women in Denmark.

**WHAT IS KNOWN ALREADY:**

During the study period 1994–2017, three ‘fresh’ IVF or ICSI treatments with hormonal stimulation were offered free of charge in public healthcare in Denmark, while additional ART treatments were self-financed in the private healthcare sector. Earlier research indicates that higher socioeconomic position (SEP) increases the probability of initiating ART treatment; however, knowledge about SEP and its relation to outcomes after ART treatment is limited.

**STUDY DESIGN, SIZE, DURATION:**

This is a national, register-based cohort study based on the Danish National ART-Couple (DANAC II) Cohort. Women initiating ART treatment during 1994–2017 were identified in the Danish IVF register and individually linked with data from sociodemographic registries and the Medical Birth Registry using the Danish Personal Identification number. The study population comprised 68 738 women who initiated ART. The women were followed until achieving a live birth, death, migration, or end of study (31/12/2017). The median follow-up time was between 1.3 and 1.9 years.

**PARTICIPANTS/MATERIALS, SETTINGS, METHODS:**

Women included in the analyses were aged 18–45 years. Overall associations between achieving a live birth after initiating ART treatment and educational level, labour market attachment, and income were examined and further stratified by nulliparity at baseline and age group. Hazard ratios (HRs) with confidence intervals (CIs) were estimated by Cox proportional hazards regression, and with adjustment for potential confounders including age and ethnicity. Live births refer to both treatment and non-treatment-related births.

**MAIN RESULTS AND THE ROLE OF CHANCE:**

Adjusted results showed that educational level, labour market attachment, and income significantly determined the probability of achieving a live birth after initiating ART treatment. HRs of achieving a live birth after initiating ART treatment increased stepwise with higher education and income level, respectively. Compared with primary school education, the highest HR was found among women with a research education (adjusted HR (aHR) = 3.02 [95% CI, 2.71; 3.35]). Unemployed women had the lowest HR of achieving a live birth after initiating ART treatment (aHR = 0.67 [95% CI, 0.64; 0.69]) compared to employed women. Women at the highest income level had two times higher HR (aHR = 2.00 [1.94; 2.06] of achieving a live birth after initiating ART compared to women at the lowest income level. Analyses of different age groups showed that higher SEP was associated with a live birth after ART treatment in all ages. The same consistency was found for both childless women and women who had a child or children prior to ART treatment. Analyses showed that women with higher SEP were more likely to continue ART treatment after unsuccessful attempts, which could explain the observed social inequality in achieving a live birth after ART treatment.

**LIMITATIONS, REASONS FOR CAUTION:**

The Danish IVF register did not register non-ART treatments (intrauterine inseminations) before 2007; thus, these treatments were not included. Whether the severity of infertility differs among SEP groups is unclear. Also, geographical distance to fertility clinics could have provided additional perspectives.

**WIDER IMPLICATIONS OF THE FINDINGS:**

Free access to fertility treatment in public healthcare should ideally provide equal access. Patients encounter barriers during treatment, and as shown, treatment success differs based on socioeconomic background. Difficulties encountered during treatment that challenge certain patients more than others based on their socioeconomic background need to be considered further.

**STUDY FUNDING/COMPETING INTEREST(S):**

No specific funding was achieved for this project. The authors have no conflicts of interest to declare.

**TRIAL REGISTRATION NUMBER:**

N/A

WHAT DOES THIS MEAN FOR PATIENTS? Reproductive diseases impact millions of women and men globally. Around 18% of women and men of reproductive age experience infertility—that a pregnancy has not been achieved after more than one year of trying. In the Nordic countries, fertility treatment is available in the public healthcare sector and at private fertility clinics. Until 2024, the Danish public healthcare system offered access to up to three treatment cycles with IVF or ICSI, where the treatment costs were financed by the state. Women/couples also have access to fertility treatment at private clinics, where the patients pay all treatment costs out-of-pocket. Since 2007, single women and same-sex female couples have also had access.

This study is a national register-based study including all women/couples having received fertility treatment in Denmark from 1994 to 2017. Denmark is one of the countries in the world with the highest number of fertility treatment cycles, and 13% of all children are born after fertility treatment. Despite access to fertility treatment in the public healthcare system, fewer women with a lower income and shorter education are seeking fertility treatment compared to women with higher income and longer education.

In this study, we found that more women with higher income, longer education, and employment achieved a live birth after undergoing fertility treatment. Also, we found that more of these women proceeded with fertility treatment after one or more unsuccessful treatment cycles. Hence, there are socioeconomic differences among fertility patients that could indicate practical and emotional barriers needing attention from clinicians. Practical barriers could include challenges related to non-flexible work hours and longer geographical distances to a fertility clinic.

## Introduction

Infertility is a frequent chronic disease in women and men of reproductive age. A recent review and meta-analysis found that the lifetime prevalence of infertility worldwide was approximately 17.5%, while the point prevalence was found to be 12.6% (Cox *et al.*, 2022). The use of ART treatment has steadily increased since the birth of the first child conceived by IVF in 1978, particularly in high-income countries. Denmark is one of the countries in the world with the easiest accessibility to ART treatment. In 2020, Denmark conducted 12 921 ART treatment cycles per million reproductive-aged women (15–45 years), while the average number of ART treatment cycles for European countries was 8808 (Smeenk *et al.*, 2025).

The general trend shows a decline in the number of children born in Denmark during the study period, from 1809 children born per 1000 women in 1994 to 1752 children in 2017 (Statistics Denmark, 2024a). The number of children born in Denmark has declined further from 2017 to 2024 (1465 children born per 1000 women in 2024), which corresponds to an increased use of fertility treatment. In 2022, children conceived through medically assisted reproduction (MAR) accounted for 13.0% of the national birth cohort, of which 9.5% were conceived using ART (Danish Fertility Society, 2022; Statistics Denmark, 2024b). Healthcare in Denmark is primarily publicly funded through taxation; nevertheless, private fertility clinics requiring out-of-pocket payment constitute an important sector providing approximately half of all ART treatments (Danish Health Data Authority, 2021).

In Denmark, no associations have been identified between infertility and sociodemographic or economic factors (Schmidt *et al.*, 1995; Eliasen *et al.*, 2024), but one study found that completion of at least 9 years of schooling was significantly associated with seeking medical help for infertility (Schmidt *et al.*, 1995). Socioeconomic position (SEP) and its association with initiating first ART treatment in the public and private healthcare sectors have been studied in the Danish cohort also used in the present study (Brautsch *et al.*, 2023), showing that women with higher SEP were consistently more likely to initiate ART treatment, with SEP defined as educational level, labour market attachment, and income level (Brautsch *et al.*, 2023).

An extensive literature review about whether socioeconomic factors influence the outcome of ART treatments revealed surprisingly few studies focusing on the association between SEP and achieving a live birth after initiating treatment (Imrie *et al.*, 2023). However, few population studies have demonstrated that women conceiving and giving birth after ART treatment were more likely to be in the highest SEP groups when compared to women giving birth without ART, regardless of whether education, income, or occupation was used as the marker for SEP (Räisänen *et al.*, 2013; Barbuscia *et al.*, 2019; Tierney and Cai, 2019; Goisis *et al.*, 2020, 2024). These studies do not allow us to determine whether the lower numbers of children conceived through ART among more disadvantaged individuals are due to lower ART success rates, reduced demand for ART services, or barriers to accessing treatment.

The existing literature that focused on whether SEP influenced ART treatment outcomes such as pregnancy or live birth, is cross-sectional studies, which, in terms of the study design, did not take time, censoring, or competing risks into account (Källén *et al.*, 2005; Barzilai-Pesach *et al.*, 2006; Mahalingaiah *et al.*, 2011; Smith *et al.*, 2011; Richardson *et al.*, 2020). Two studies from the United States and one from Israel did not find associations between SEP and achieving a live birth (Mahalingaiah *et al.*, 2011) or a pregnancy (Barzilai-Pesach *et al.*, 2006; Smith *et al.*, 2011) after initiating ART treatment.

Findings from a recent study suggest that residing in low SEP neighbourhoods is associated with a reduced likelihood of achieving an ongoing pregnancy within 2.5 years after starting IVF treatment. Furthermore, this association also extends to live birth rates (Speksnijder *et al.*, 2024). Furthermore, a multinational register-based study, including Danish data, recently found that university-educated mothers are more likely to give birth after assisted reproduction compared with mothers with lower levels of education. The study also found that families with a higher income were more likely to have a child after using MAR (Goisis *et al.*, 2024). In line with this, a previous register-based study from Denmark showed that among childless women, patients with at least a college degree were 24% more likely to obtain a live birth at the first ART cycle compared to women not having a high school degree (Groes *et al.*, 2017). This study covered the period 1995–2009, and included only nulliparous, cohabiting/married women aged 25–45 years. As legislation in Denmark since 2007 has permitted single women and same-sex female couples to receive MAR, our study will expand on previous studies. In our latest year of data inclusion, 2017, 8% of all live births after ART were among single mothers (Danish Fertility Society, 2017). It is of clinical importance to include the full possible ART patient population.

Hence, our study assessing the association between SEP and achieving a live birth after initiating ART treatment used data from a nationwide Danish register-based cohort of 18–45-year-old women, being single/married/cohabiting, and undergoing ART treatment from 1994 to 2017. The primary aim was to examine the association between socioeconomic factors, including education level, labour market attachment and income level, and achieving a live birth following ART treatment initiation among 18- to 45-year-old women in Denmark. The secondary aim was to assess the association between SEP and continuation of ART treatment among women who had not yet achieved a live birth.

## Materials and methods

### Ethical approval

This project was approved by the Danish Data Protection Agency (The Faculty of Health and Medical Sciences, University of Copenhagen, SUND-2017-34; project number 514-0279/19-3000) and by The Danish Health Data Authority (FSEID-00003042, 3084, 2554, 3043, 3086). According to the Danish legislation, solely register-based studies do not require approval from a scientific ethical committee or patient informed consent, because these studies do not involve direct contact with persons.

### Setting

Since 1994, all ART treatments performed in public and private clinics in Denmark have been recorded in the IVF Registry. During the study period, women aged 18–40 years were eligible for up to three ‘fresh’ ART cycles (IVF or ICSI) with hormonal stimulation financed by the public healthcare system for a couple’s first child. Since 2007, childless single women and female same-sex couples have also had access to ART treatment at public and private fertility clinics. Women up to 46 years of age can access ART treatment at private clinics regardless of whether they already have a child. In the public healthcare system, patients must pay for their medication, while the treatment cost itself is free. Medication costs for a single ‘fresh’ ART treatment with hormonal stimulation is around 10–12 000 DKK (∼1500 EUR). In Denmark, the annual out-of-pocket costs of medicine cannot exceed 4735 DKK (∼640 EUR) (2025 prices; Danish Medicines Agency, n.d.). If a patient in ART treatment already has reached this level (e.g. due to medication for other diseases), the ART medication is fully reimbursed.

### Study population

The Danish National ART-Couple II (DANAC II) Cohort consists of all women undergoing ART-treatment during 1994–2017 and who were registered in the Danish IVF register. Due to the Danish Health Authorities’ re-organization of national health registers, access to IVF data only up to 2017 was available when we conducted the study. In the present study, a woman was considered exposed from the date of first initiated ART treatment. The study population consisted of 68 738 women treated with ART. The women were aged 18–45 years. In the present study, ART treatment consists of treatment with IVF or ICSI. Furthermore, live births were included regardless of whether they were treatment or non-treatment-related pregnancies after initiating ART treatment.

### Data

Data on first ART treatment in either the public or private sector were obtained from the national Danish IVF register, as well as the number of subsequent ART treatments before achieving a live birth, whether it was treatment related or non-treatment related. The ART treatments included fresh IVF and ICSI cycles with hormone stimulation, with reference to how access to treatment is regulated in the public healthcare system (i.e. three fresh cycles reimbursed). Consequently, cycles with frozen embryo transfer were not counted. Date of childbirth after initiating ART was obtained from the Medical Birth Register. Information on whether the women had children before seeking the first treatment was also obtained from the Medical Birth Register. Data on educational level, labour market attachment, income, marital status, age, and origin were retrieved for relevant timepoints from sociodemographic registries accessed via Statistics Denmark. The Danish Personal Identification number enabled data linkage between registers on an individual level. The SEP and covariate register information were obtained at the time of initiating ART treatment for each woman.

Educational level was categorized as primary school, high school, vocational education, short higher education (vocational academy education, 2–2.5 years), medium higher education (professional bachelor’s degree and bachelor’s degree, 3–4.0 years), long higher education (master’s degree, 5–6 years), and research education (PhD). Labour market attachment was categorized as whether the woman was part of the labour force as either employed or unemployed, or whether she was outside the labour force, i.e. due to sickness pay, leave benefit, or early retirement. Students were recorded as a separate group. Income was categorized as an equivalized annual household income as a relative measure, divided into quintiles. Due to a lack of information from sociodemographic registries from 1994, women who initiated ART treatment in 1994 were registered with information from 1995.

Marital status was categorized as married, cohabitant, and single/non-cohabiting. Origin was recorded as Danish origin, immigrant, or descendant. Danish origin was defined as persons with at least one parent born in Denmark with Danish citizenship, immigrant was defined as persons born in a foreign country to parents of whom none are Danish citizens or born in Denmark, and descendant was defined as persons born in Denmark to parents of whom none are Danish citizens or born in Denmark.

### Statistical analyses

For descriptive purposes, the frequency of undergoing ART treatment was grouped by education level, labour market attachment, income level, and investigated according to marital status, origin, age at first ART treatment, child/children prior to first ART treatment (nulliparity at baseline) and public/private clinic at first ART treatment.

Directed acyclic graphs (DAGs) were used to identify confounders in the association between socioeconomic factors and achieving a live birth after initiating ART treatment (Shrier and Platt, 2008). DAGs serve as a tool to distinguish between potential confounding and mediating factors, and to plan analyses according to the assumptions made. One guiding principle is to avoid adjustment for mediating factors. Age and origin were identified as potential confounders as they were associated with socioeconomic factors and with achieving a live birth. Marital status, having at least one child prior to ART, the number of ART treatments, and public or private fertility clinics at first ART treatment were identified as mediators and were not included in the analyses (Shrier and Platt, 2008). This approach was chosen because the total effect is captured in a model solely adjusted for confounding factors, while adjustment for mediators would theoretically remove the parts of the association that work through the relevant mechanism/variable.

Overall associations between achieving a live birth after initiating ART treatment and educational level, labour market attachment, and income level were examined. Hazard ratios (HRs) with 95% confidence intervals (CIs) were estimated by the Cox proportional hazards model. Participants’ time at risk began at the time of first ART treatment initiation. Time at risk was terminated by the occurrence of the outcome of childbirth or by censoring at the event of death, migration, or end of follow-up on 31 December 2017. Death was considered a potential competing risk in all analyses, and the cumulative incidence of death before age 50 years across SEP strata was examined. Age was the underlying timescale in the Cox regression model, hence adjustment for age was inherent in all analyses. The multivariable models were further adjusted for origin. Given that SEP indicators are highly correlated and the effect of one variable is not easily distinguished from another (Green and Popham, 2019, Tilling *et al.*, 2013), we chose the combined approach of having each SEP indicator in a separate model adjusted for confounders as the main results, and additionally include a ‘mutually adjusted’ model, in which adjustments were made for the other two SEP indicators as if they were confounders. The analyses were further stratified by nulliparity at baseline in multivariate analyses. With a focus on trends across strata, reference groups were selected as the lowest educational group (primary school) and the lowest income group (the lowest quintile). For employment status, the reference group selected was employed. Educational level and income were additionally tested with the middle group (short higher education and middle-income quintile) as a reference to enable reporting of estimates for the highest and lowest groups relative to the middle group.

A secondary analysis using a Cox regression model was made to investigate the association between SEP and the likelihood to continue ART treatment after unsuccessful attempts, given that this factor majorly impacts the chance of achieving a live birth. The secondary analyses were set up to include treatments that could potentially be covered by public healthcare (attempt 1–3 fresh cycle) as well as treatments that are beyond public coverage and thus would be self-financed (attempt 4–5 fresh cycle), as this could enhance the impact of SEP on progressing to a next treatment attempt. Additional considerations for these analyses were that patient characteristics change from first to second ART treatment (and so on), both due to some achieving a live birth and others terminating treatment for a variety of reasons, and meaningful comparisons between these groups can be difficult. It appears that women initiating more treatments might be healthier, and studies have observed a lower risk of death (Vassard *et al.*, 2018) and a lower risk of depression with an increasing number of ART treatments (Sejbæk *et al.*, 2015). With this complexity in mind, the analyses of continuation of ART treatment before achieving a live birth was conducted as four separate analyses, including those having gone through a treatment attempt (first to fourth attempt), being at risk of progressing to the next attempt (second to fifth attempt), which was defined as the outcome. Inclusion criteria for every analysis were that no live birth had been achieved after treatment initiation. Censoring was conducted due to the first occurring event of either live birth, death, migration or end of follow-up.

Missing data were handled using an available-case approach, i.e. pairwise deletion. Any observations containing missing values in all socioeconomic variables (education level, labour market attachment, and income) in the analyses were excluded. Missing values in one socioeconomic variable were excluded only from the analyses regarding that particular determinant. Since the data used in this study are collected from population registers and in healthcare administration independent of the research question, we consider it fair to assume that they are missing at random. As a sensitivity analysis, the multivariable analysis was repeated, including marital status, nulliparity at baseline, and public or private clinic at first ART treatment as potential confounders. Furthermore, considering the expansion of treatment access that was implemented in the public healthcare system in 2007, separate sensitivity analyses were conducted for the period 2007–2017.

All statistical analyses were performed in SAS 9.4 (SAS Institute Inc., SAS software, Version 9.4. Cary, NC, USA).

## Results

A total of 68 738 women had received a first fresh ART treatment during 1994–2017. Most of the population were married or cohabitants (46 and 40%), received first ART treatment at a public fertility clinic (86%) and did not have children prior to initiating treatment (79%). The percentage of missing observations for the socioeconomic factors were <0.5% for income and labour market attachment and approximately 2% for educational level.

### Educational level

Mean age at first ART treatment was higher among women with a research education (37.1, SD 4.0) than among women with primary school (31.9, SD 5.2), and a larger proportion of women with the two highest education levels achieved their first ART treatment at a private fertility clinic (17–19% vs. 10%). Origin differed across education levels. The largest proportion of immigrants or descendants was found among women with a research education (32%), while the smallest proportion was women with either primary school or long higher education (6%). Only 16% of women with a high school education had one or more children prior to ART treatment, while the proportion of parous women was largest among women with primary school (29%) or research education (25%) (Table 1).

**Table 1.** Population characteristics of women initiating ART treatment at age 18–45 years distributed by education, labour market attachment, and income (The DANAC II cohort 1994–2017).

	Education, n (%) *(missing = 1497)*						
	Primary school	High school	Vocational education	Short higher education	Medium higher education	Long higher education	Research education
**Total**	8000 (12)	6425 (9)	20 817 (31)	3798 (6)	18 714 (28)	8907 (13)	580 (1)
**Marital status**							
Married	3313 (41)	3309 (51)	9733 (47)	1843 (49)	8340 (45)	3955 (44)	303 (52)
Cohabitant	3336 (42)	2244 (35)	8742 (42)	1520 (40)	7666 (41)	3367 (38)	171 (30)
Single/non-cohabitant	1348 (17)	872 (14)	2340 (11)	434 (11)	2707 (14)	1585 (18)	106 (18)
*Missing = 249*							
**Origin**							
Danish origin	7524 (94)	4592 (71)	19 168 (92)	3339 (88)	16 638 (89)	8333 (94)	396 (68)
Immigrant or descendant*	475 (6)	1833 (29)	1649 (8)	459 (12)	2076 (11)	574 (6)	184 (32)
*Missing = 56*							
**Age at first ART treatment**							
Mean (± SD)	31.9 (± 5.2)	32.1 (± 5.4)	32.5 (± 4.6)	33.7 (±4.4)	33.3 (± 4.5)	35.1 (±4.2)	37.1 (± 4.0)
[5% min; 95% max]	[23.4; 40.1]	[23.8; 40.9]	[25.5; 40.3]	[27.1; 41.2]	[26.9; 41.4]	[29.0; 42.3]	[30.4; 43.4]
**Child/children before ART treatment**							
No	5688 (71)	5366 (84)	16 206 (78)	3083 (81)	15 062 (80)	7015 (79)	437 (75)
Yes	2312 (29)	1059 (16)	4611 (22)	715 (19)	3652 (20)	1892 (21)	143 (25)
**Public/private fertility clinic at first ART treatment**							
Public fertility clinic	7183 (90)	5534 (86)	18 364 (88)	3280 (86)	16 149 (86)	7202 (81)	480 (83)
Private fertility clinic	817 (10)	891 (14)	2453 (12)	518 (14)	2565 (14)	1705 (19)	100 (17)

	Labour market attachment, n (%)**				Income, n (%)***				
	Employed	Outside the workforce	Unemployed	Students	1. Quintile (lowest)	2. Quintile	3. Quintile	4. Quintile	5. Quintile (highest)
**Total**	57 201 (83)	4106 (6)	4505 (7)	2924 (4)	13 721 (20)	13 722 (20)	13 722 (20)	13 722 (20)	13 722 (20)
**Marital status**									
Married	26 238 (46)	2226 (57)	2045 (46)	1151 (39)	6433 (47)	6746 (49)	6526 (48)	5947 (43)	6002 (44)
Cohabitant	23 317 (41)	1033 (26)	1593 (35)	1333 (46)	4184 (31)	5241 (38)	5523 (40)	6136 (45)	6192 (45)
Single/non-cohabiting	7571 (13)	686 (17)	856 (19)	438 (15)	3031 (22)	1719 (13)	1656 (12)	1628 (12)	1511 (11)
*Missing = 249*									
**Origin**									
Danish origin	52 004 (91)	2521 (62)	3354 (74)	2424 (83)	10 766 (79)	12 324 (90)	12 398 (90)	12 523 (91)	12 228 (89)
Immigrant	4663 (8)	1505 (37)	1102 (25)	452 (15)	2784 (20)	1287 (9)	1199 (9)	1083 (8)	1360 (10)
Descendant	506 (1)	52 (1)	49 (1)	48 (2)	171 (1)	110 (1)	124 (1)	116 (1)	134 (1)
*Missing = 56*									
**Age at first ART treatment**									
Mean (± SD)	33.4 (±4.6)	33.4 (± 5.5)	33.0 (±5.1)	29.8 (±4.8)	32.5 (± 5.1)	32.5 (±4.7)	32.9 (±4.7)	33.4 (±4.6)	34.7 (± 4.4)
[5% min; 95% max]	[26.1; 41.1]	[24.2; 42.5]	[24.7; 41.2]	[23.2; 39.2]	[24.1; 40.8]	[25.3; 40.3]	[25.8; 40.7]	[26.3; 41.4]	[27.7; 42.1]
**Child/children before ART treatment**									
No	45 215 (79)	3079 (75)	3193 (71)	2561 (88)	9890 (72)	10 651 (78)	10 983 (80)	11 205 (82)	11 201 (82)
Yes	11 986 (21)	1027 (25)	1312 (29)	363 (12)	3831 (28)	3071 (22)	2739 (20)	2517 (18)	2521 (18)
**Public/private fertility clinic at first ART treatment**									
Public fertility clinic	49 331 (86)	3534 (86)	3919 (87)	2673 (91)	11 688 (85)	11 825 (86)	11 942 (87)	12 139 (88)	11 775 (86)
Private fertility clinic	7870 (14)	572 (14)	586 (13)	251 (9)	2033 (15)	1897 (14)	1780 (13)	1583 (12)	1947 (14)

* Immigrants and descendants are combined in one group due to few persons in each stratum.

** Labour market attachment was recorded as either employed, unemployed, or outside labour market attachment due to sickness pay, education allowance, leave benefit, or early retirement. Students were recorded as a separate group.

*** Income was recorded as an equivalized annual household income used as a relative measure divided into quintiles.

### Labour market attachment

Mean age at first ART treatment was approximately 33.0–33.4 years for both employed and unemployed women, as well as women outside the workforce, while students were younger (mean age 29.8, SD 4.8). The proportion of immigrants was significantly higher for unemployed women (37%) and women outside the workforce (25%) than for employed women (8%) (Table 1). The largest proportion of women with children prior to ART treatment was unemployed (29%). In contrast, 21% of employed women had one or more children prior to treatment.

### Income

Mean age at first ART treatment increased with higher income level (lowest income level 32.5, SD 5.1 vs. highest income level 34.7, SD 4.4 years). The proportion of women who were single/non-cohabiting was similar across the income quintiles (11–13%), except for women in the lowest income quintile (22%). Further, the proportion of women with an immigrant background was considerably higher in the lowest income quintile than in the quintiles above (20% vs. 8–10%). Among women with the lowest income level, 28% had one or more children prior to the treatment, which contrasted with women with higher income levels (18–22%) (Table 1).

### Follow-up analyses

The median follow-up time according to SEP groups was between 1.3 (0.7–18.2) years and 1.9 (0.7–22.3) years (Table 2). A larger proportion of women with higher education and income levels achieved a live birth after initiating ART treatment (5041 (63%) primary school vs. 6620 (74%) longer higher education; 8956 (65%) lowest income level vs. 9700 (71%) highest income level). The same tendency was seen for labour market attachment (2404 (59%) women outside the workforce vs. 41 025 (72%) employed women) (Table 2).

**Table 2.** Association between socioeconomic position and first livebirth after initiation of ART analyzed in Cox regression reported as HR with 95% CI (The DANAC II cohort 1994–2017).

	Follow up, years	Events	Model 1^a^	Model 2^b^	Model 3^c^
	Median (5–95% IQR)	n (%)	HR [95% CI]	HR [95% CI]	HR [95% CI]
**Education**					
Primary school	1.9 (0.7–22.3)	5041 (63)	1	1	1
High school	1.5 (0.7–18.7)	4506 (70)	1.45 [1.39; 1.51]	1.52 [1.46; 1.58]	1.40 [1.34; 1.46]
Vocational education	1.5 (0.7–20.2)	14 667 (70)	1.41 [1.36; 1.46]	1.41 [1.37; 1.46]	1.29 [1.25; 1.34]
Short higher education	1.4 (0.7–17.8)	2738 (72)	1.80 [1.72; 1.89]	1.82 [1.74; 1.91]	1.55 [1.47; 1.62]
Medium higher education	1.3 (0.7–18.2)	13 713 (73)	1.97 [1.91; 2.04]	2.00 [1.93; 2.06]	1.70 [1.64; 1.76]
Long higher education	1.3 (0.7–14.2)	6620 (74)	2.59 [2.49; 2.69]	2.59 [2.50; 2.69]	1.99 [1.91; 2.07]
Research education	1.3 (0.7–12.5)	368 (63)	2.88 [2.59; 3.20]	3.02 [2.71; 3.35]	2.30 [2.04; 2.53]
**Labour market attachment** *					
Employed	1.4 (0.7–19.7)	41 025 (72)	1	1	1
Outside the workforce	1.6 (0.7–19.4)	2404 (59)	0.75 [0.72; 0.78]	0.78 [0.75; 0.81]	1.02 [0.98; 1.07]
Unemployed	1.9 (0.7–21.8)	2752 (61)	0.66 [0.63; 0.68]	0.67 [0.64; 0.69]	0.90 [0.86; 0.93]
Student	1.3 (0.7–14.3)	2199 (75)	0.92 [0.89; 0.97]	0.93 [0.89; 0.97]	1.05 [1.00; 1.10]
**Income** **					
1. Quintile (lowest)	1.7 (0.7–22.3)	8956 (65)	1	1	1
2. Quintile	1.6 (0.7–21.7)	9632 (70)	1.12 [1.10; 1.16]	1.11 [1.08; 1.14]	1.04 [1.01; 1.07]
3. Quintile	1.5 (0.7–18.6)	9986 (73)	1.37 [1.33; 1.41]	1.35 [1.31; 1.39]	1.18 [1.15; 1.22]
4. Quintile	1.4 (0.7–13.4)	10 074 (73)	1.67 [1.63; 1.72]	1.65 [1.61; 1.70]	1.36 [1.32; 1.41]
5. Quintile (highest)	1.3 (0.7–10.2)	9700 (71)	2.02 [1.96; 2.08]	2.00 [1.94; 2.06]	1.53 [1.48; 1.58]

a Univariate analysis adjusted for age due to underlying time variable.

b Multivariate analysis adjusted for age and origin.

c Multivariate analysis adjusted for age and origin and mutual adjustment for education, labour market attachment and income.

* Labour market attachment was recorded as either employed, unemployed, or outside labour market attachment due to sickness pay, education allowance, leave benefit, or early retirement. Students were recorded as a separate group.

** Income was recorded as an equivalized annual household income used as a relative measure divided into quintiles.

HR, hazard ratio; IQR, interquartile range.

Women with higher educational levels, employed women, and women with higher income had higher HRs of achieving a live birth after initiating ART treatment. Women with a research education had a higher HR of achieving a live birth after initiating ART treatment than women with primary school education (aHR = 3.02 [95% CI, 2.71; 3.35]), women outside the workforce had a lower HR of achieving a live birth after initiating ART treatment than employed women (aHR = 0.75 [95% CI, 0.72; 0,78]), and women at the highest income level had two times higher HR of achieving a live birth after initiating ART treatment than women at the lowest income level (aHR = 2.00 [95% CI, 1.94; 2.06]). HR’s for achieving a live birth increased for every stepwise increase in income and educational level (Table 2).

The association between labour market attachment and achieving a live birth after initiating ART treatment became less significant when adjusting for education level, income level, age, and origin. Hence, women outside the workforce no longer differed significantly from employed women (aHR = 1.02 [95% CI, 0.98; 1.07]) (Table 2, Model 3).

When the adjusted model was furthermore adjusted for marital status, nulliparity at baseline, and whether the first ART treatment was initiated at a public or private fertility clinic, the analysis showed similar results as presented in Table 2, Model 2. Analyses including only ART treatment initiation during 2007–2017, when single women and female same-sex couples were allowed to use ART treatment, showed similar tendencies, however, with slightly lower HRs (Supplementary Table S1).

Repeating Model 2 analyses with the middle educational level as reference, the aHRs were 0.55 [95% CI, 0.52; 0.58] and 1.66 [95% CI, 1.48; 1.85] for the lowest and highest educational level, respectively. For income analyses with the middle quintile as reference, the aHRs were 0.74 [95% CI, 0.72; 0.76] and 1.48 [95% CI, 1.44; 1.53] for the lowest and highest income quintile, respectively.

#### Analysis by nulliparity

Most of the population did not have children prior to ART treatment (79%). Significant associations between level of education, labour market attachment, and income level, and achieving a live birth after initiating ART treatment were still present in both groups (women without/with child or children prior to ART treatment) (Table 3). Nevertheless, the association between SEP and achieving a live birth after initiating ART treatment was slightly more pronounced for women with one or more children prior to ART compared to the main analysis based on the total population. However, the same tendencies as in the main analysis (Table 2, Model 2) were found whether the woman had one or more children before ART treatment (nulliparous/parous). HR of achieving a live birth after ART treatment increased with incremental education and income level for both nulliparous and parous women (Table 3). For both women with and without children before ART treatment, unemployed women had the lowest HR for achieving a live birth compared to employed women (parous: aHR = 0.61 [95% CI, 0.56; 0.66]; nulliparous: aHR = 0.68 [95% CI, 0.65; 0.71]) (Table 3).

**Table 3.** Association between socioeconomic position and first child after ART initiation categorized by women with and without children before first ART initiation analyzed in Cox regression reported in HR with 95% CI (The DANAC II cohort 1994–2017).

	Model 2*					
	Women without child/children prior to ART n = 54 050			Women with child/children prior to ART n = 14 688		
	Follow up, years Median (5–95% IQR)	Events n (%)	HR [95% CI]	Follow up, years Median (5–95% IQR)	Events n (%)	HR [95% CI]
**Education**						
Primary school	1.79 (0.71–22.66)	3754 (66)	1	2.36 (0.71–21.60)	1287 (56)	1
High school	1.47 (0.71–18.52)	3883 (72)	1.55 [1.48; 1.62]	1.77 (0.72–18.98)	623 (59)	1.49 [1.35; 1.65]
Vocational education	1.47 (0.71–20.27)	11 965 (74)	1.45 [1.39; 1.50]	1.79 (0.71–20.13)	2702 (59)	1.33 [1.25; 1.43]
Short higher education	1.38 (0.71–17.74)	2312 (75)	1.88 [1.78; 1.98]	1.56 (0.73–18.12)	426 (60)	1.68 [1.50; 1.87]
Medium higher education	1.33 (0.71–17.87)	11 384 (76)	2.02 [1.95; 2.10]	1.42 (0.71–19.04)	2329 (64)	1.96 [1.83; 2.10]
Long higher education	1.26 (0.71–13.84)	5352 (76)	2.58 [2.47; 2.69]	1.33 (0.71–15.54)	1268 (67)	2.79 [2.57; 3.01]
Research education	1.30 (0.66–11.84)	289 (66)	2.98 [2.64; 3.36]	1.33 (0.65–13.40)	79 (55)	3.34 [2.66; 4.20]
**Labour market attachment** **						
Employed	1.39 (0.71–19.70)	33 611 (74)	1	1.55 (0.71–19.70)	7414 (62)	1
Outside the workforce	1.57 (0.64–18.97)	1863 (61)	0.77 [0.73; 0.81]	1.86 (0.69–20.65)	541 (53)	0.81 [0.75; 0.89]
Unemployed	1.78 (0.71–21.88)	2084 (65)	0.68 [0.65; 0.71]	2.62 (0.71–21.66)	668 (51)	0.61 [0.56; 0.66]
Students	1.24 (0.70–14.02)	1978 (77)	0.95 [0.91; 1.00]	1.45 (0.71–16.05)	221 (61)	0.77 [0.67; 0.88]
**Income** ***						
1. Quintile (lowest)	1.56 (0.70–22.29)	6829 (69)	1	2.35 (0.71–22.29)	2127 (56)	1
2. Quintile	1.54 (0.71–21.97)	7734 (73)	1.07 [1.04; 1.11]	1.69 (0.71–20.76)	1898 (62)	1.27 [1.19; 1.35]
3. Quintile	1.43 (0.72–18.88)	8296 (76)	1.32 [1.28; 1.37]	1.58 (0.72–16.95)	1690 (62)	1.46 [1.37; 1.56]
4. Quintile	1.34 (0.71–13.36)	8482 (76)	1.62 [1.57; 1.67)	1.45 (0.71–13.73)	1592 (63)	1.82 [1.70; 1.94]
5. Quintile (highest)	1.29 (0.71–9.51)	8165 (73)	1.93 [1.87; 2.00]	1.36 (0.71–11.70)	1535 (61)	2.42 [2.27; 2.59]

* Analysis based on main analysis (Table 2, Model 2). Adjusted for age and origin.

** Labour market attachment was recorded as either employed, unemployed, or outside labour market attachment due to sickness pay, education allowance, leave benefit, or early retirement. Students were recorded as a separate group.

*** Income was recorded as an equivalized annual household income used as a relative measure divided into quintiles.

HR, hazard ratio; IQR, interquartile range.

#### Analysis of continued ART treatments

The results from Table 4 indicated that women with higher levels of education and income are more likely to initiate subsequent ART treatment (second to fifth) after unsuccessful treatment. Women with a research education had a higher HR of initiating second ART treatment than women with primary school education (aHR = 2.41 [95% CI, 2.15; 2.70]), and women at the highest income level had over two times higher HR of initiating second ART treatment than women at the lowest income level (aHR = 2.22 [95% CI, 2.15; 2.30]). Similarly, women with a research education had almost three times higher HR of initiating fourth ART treatment (which for most people was self-financed treatment due to legislation) than women with primary school education (aHR = 2.85 [95% CI, 2.32; 3.51]). Furthermore, women at the highest income level had almost three times higher HR of initiating fourth ART treatment than women at the lowest income level (aHR = 2.89 [95% CI, 2.73; 3.06]). HR’s of initiating subsequent ART treatment after unsuccessful treatment increased for approximately every increase in educational and income level. Women outside the workforce, unemployed, and students generally had lower HR’s of initiating the subsequent ART treatment after unsuccessful treatment than employed women.

**Table 4.** Correlation between socioeconomic position and progression to next ART treatment before delivery of first livebirth after initiation of ART analysed in Cox regression reported in HR with 95% CI (The DANAC II cohort 1994–2017).

	Second ART treatment*	Third ART treatment*	Fourth ART treatment*	Fifth ART treatment*
	n = 68 738	n = 39 388	n = 23 243	n = 12 076
**Events, n (%)**	39 388 (57)	23 243 (59)	12 076 (52)	6348 (53)
**Follow up, years. Median (5–95% IQR)**	0.66 (0.16–6.83)	0.66 (0.16–10.49)	0.74 (0.16–16.09)	0.73 (0.16–15.97)
	**HR [95% CI]**	**HR [95% CI]**	**HR [95% CI]**	**HR [95% CI]**
**Education**				
Primary school	1	1	1	1
High school	1.40 [1.34; 1.46]	1.47 [1.38; 1.56]	1.49 [1.37; 1.62]	1.74 [1.55; 1.96]
Vocational education	1.37 [1.33; 1.42]	1.41 [1.35; 1.47]	1.37 [1.28; 1.46]	1.41 [1.29; 1.55]
Short higher education	1.67 [1.58; 1.75]	1.78 [1.67; 1.90]	1.84 [1.68; 2.02]	2.03 [1.78; 2.31]
Medium higher education	1.62 [1.56; 1.67]	1.81 [1.73; 1.90]	2.03 [1.90; 2.17]	2.34 [2.13; 2.57]
Long higher education	1.97 [1.89; 2.05]	2.43 [2.30; 2.56]	3.14 [2.92; 3.38]	3.87 [3.49; 4.28]
Research education	2.41 [2.15; 2.70]	2.55 [2.20; 2.95]	2.85 [2.32; 3.51]	5.30 [4.05; 6.94]
Labour market attachment**				
Employed	1	1	1	1
Outside the workforce	0.75 [0.72; 0.79]	0.83 [0.78; 0.88]	0.75 [0.69; 0.81]	0.67 [0.59; 0.76]
Unemployed	0.73 [0.70; 0.76]	0.64 [0.60; 0.67]	0.68 [0.63; 0.73]	0.67 [0.60; 0.75]
Students	0.76 [0.72; 0.80]	0.84 [0.78; 0.90]	0.87 [0.79; 0.96]	0.98 [0.86; 1.12]
**Income** ***				
1. Quintile (lowest)	1	1	1	1
2. Quintile	1.29 [1.25; 1.36]	1.19 [1.14; 1.24]	1.07 [1.00; 1.13]	1.13 [1.03; 1.23]
3. Quintile	1.50 [1.45; 1.55]	1.36 [1.31; 1.42]	1.34 [1.26; 1.42]	1.42 [1.30; 1.54]
4. Quintile	1.80 [1.75; 1.86]	1.76 [1.68; 1.83]	1.95 [1.84; 2.07]	2.25 [2.07; 2.45]
5. Quintile (highest)	2.22 [2.15; 2.30]	2.25 [2.16; 2.35]	2.89 [2.73; 3.06]	3.46 [3.19; 3.75]

* Adjusted for age and origin.

** Labour market attachment was recorded as either employed, unemployed, or outside labour market attachment due to sickness pay, education allowance, leave benefit, or early retirement. Students were recorded as a separate group.

*** Income was recorded as an equivalized annual household income used as a relative measure divided into quintiles.

HR, hazard ratio; IQR, interquartile range.

#### Cumulative incidence curves

**Figure 1.**
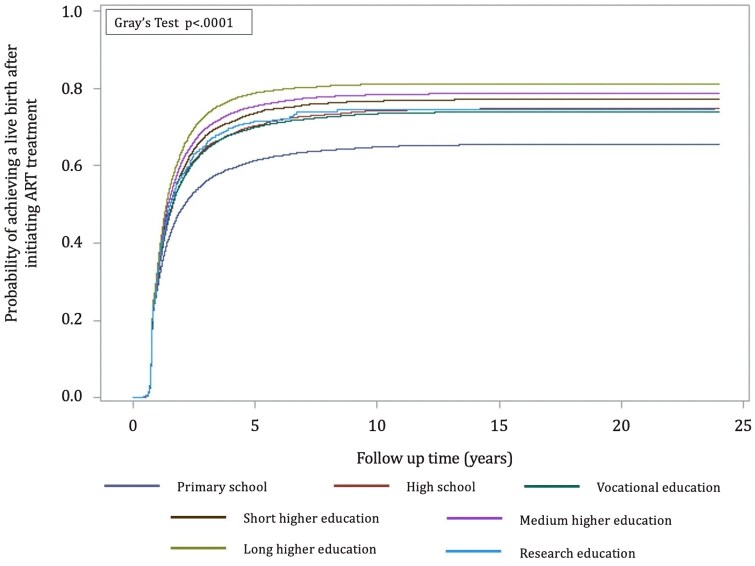
**Cumulative incidence curves indicating the probability of having a first child after starting ART treatment by educational level**.

**Figure 2.**
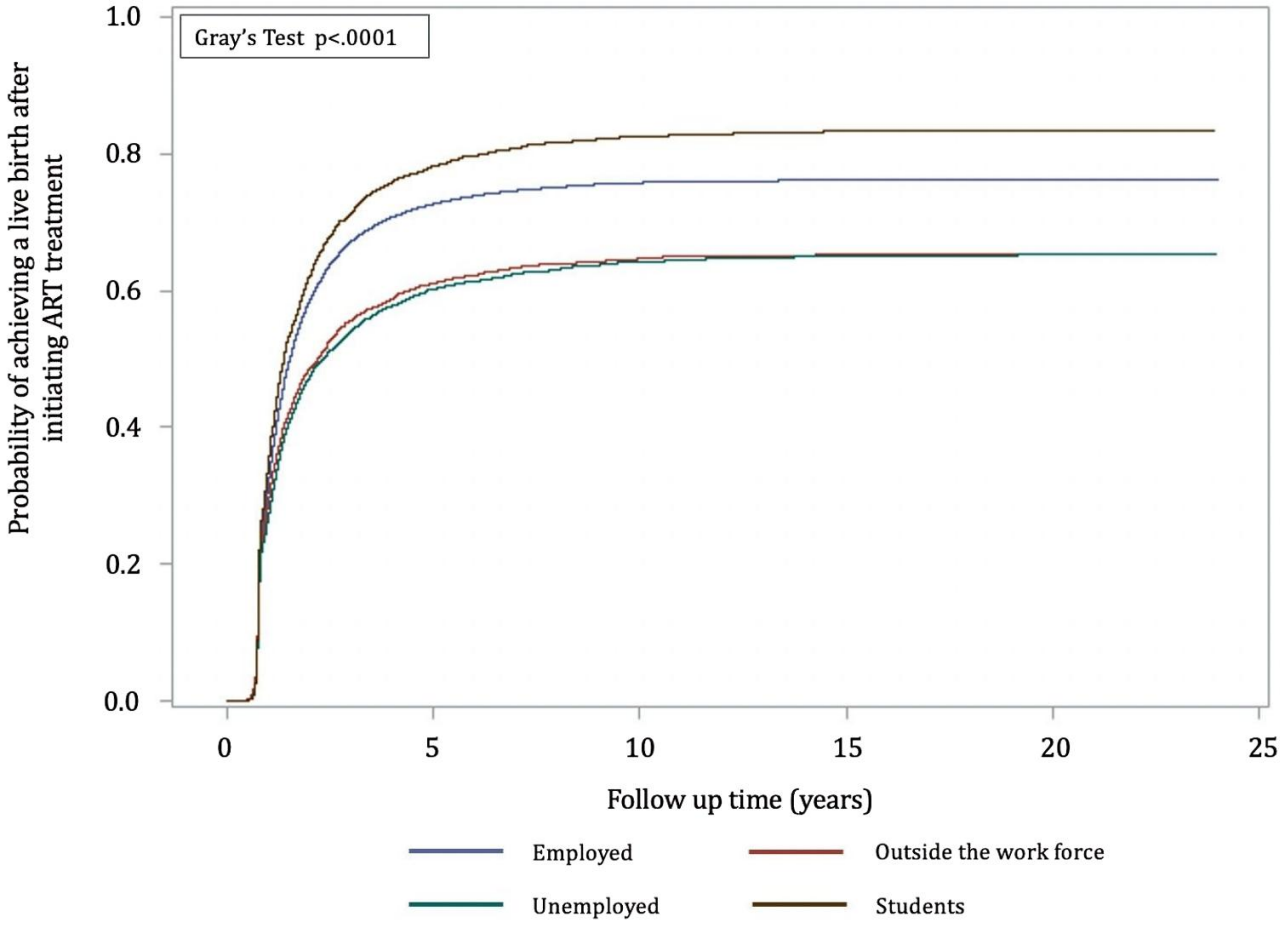
**Cumulative incidence curves indicating the probability of having a first child after starting ART treatment by labour market attachment**.

**Figure 3.**
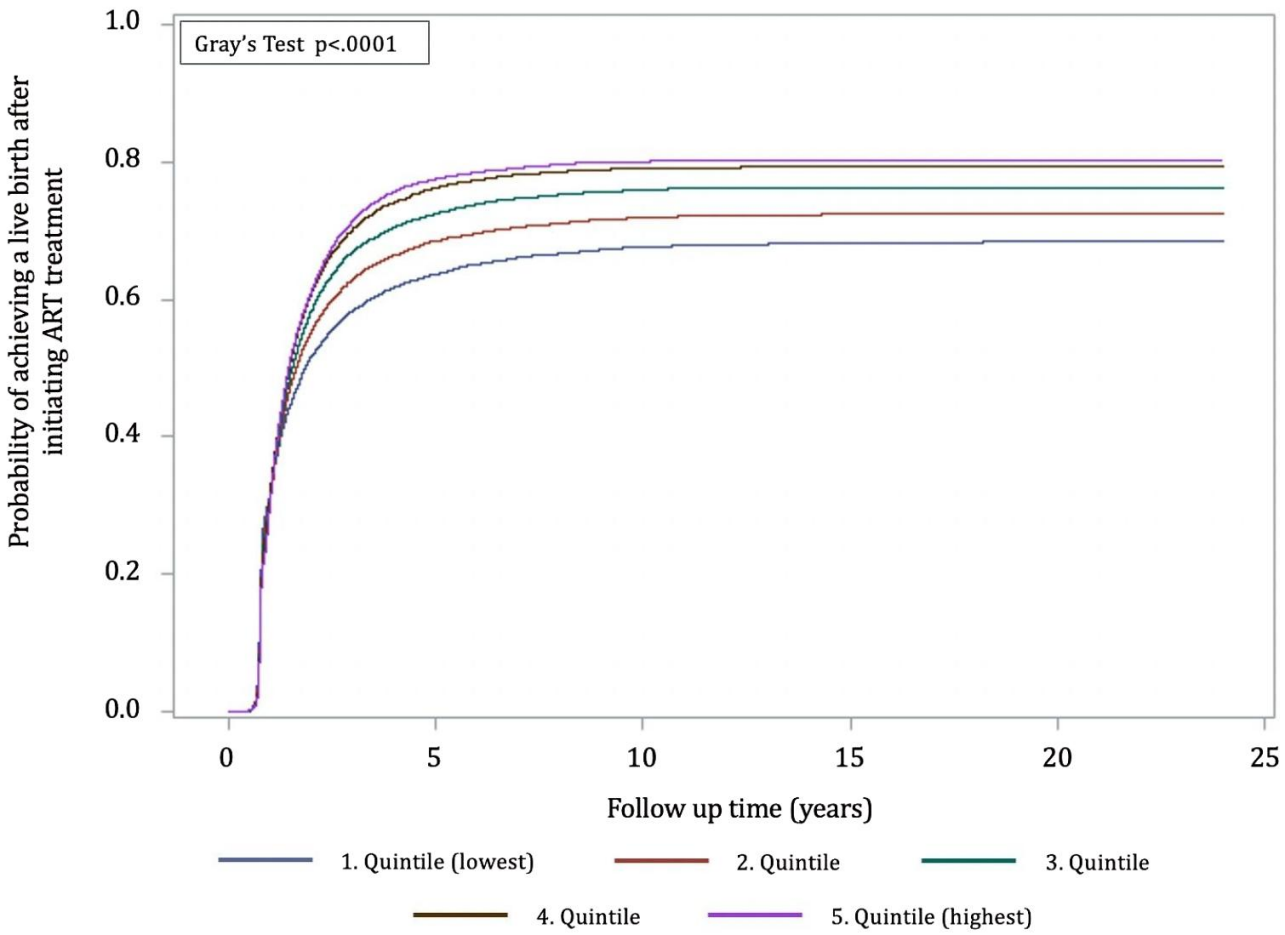
**Cumulative incidence curves indicating the probability of having a first child after starting ART treatment by income level**.

Regardless of the women’s education, labour market attachment, and income, the absolute probability of achieving a live birth after initiating ART increased in the first 5 years after the first ART treatment, after which the incidence for all groups stagnated (Figs 1, 2, and 3).

Death in the population was a rare event (<1%). Results were similar in models irrespective of incorporating death as a competing risk.

## Discussion

The results showed that the probability of achieving a first live birth after initiating ART treatment increased progressively with higher levels of education and income. Furthermore, unemployed women had the lowest likelihood of success, being a third less likely to have a live birth after ART treatment compared to employed women. Analyses also revealed that women with a higher SEP were more likely to continue ART treatment after unsuccessful attempts.

Existing literature supports the assumption that there is no difference in the prevalence of infertility according to SEP in Denmark (Schmidt *et al.*, 1995; Eliasen *et al.*, 2024), but an association between SEP and the use of ART treatment have been found (Brautsch *et al.*, 2023). In this Danish population-based sample of women, there were clear socioeconomic disparities in achieving a live birth after initiating ART treatment. Higher educational level, labour market attachment, and higher income were strongly associated with achieving a live birth after initiating ART treatment among women in Denmark.

Socioeconomic disparities in live births after ART treatment are consistent with previous studies about achieving pregnancies or live births after initiating ART treatment (Källén *et al.*, 2005; Morris *et al.*, 2011; Smith *et al.*, 2011; Murray, 2014; Groes *et al.*, 2017; Richardson *et al.*, 2020; Speksnijder *et al.*, 2024). Furthermore, the results are consistent with findings that mothers who conceived and gave birth through ART tend to have higher income and education levels (Räisänen *et al.*, 2013; Barbuscia *et al.*, 2019; Goisis *et al.*, 2020, 2024). Speksnijder *et al.* (2024) used clinical data from IVF treatments conducted between 2006 and 2020 at an IVF centre in Rotterdam. The SEP scores were based on household income, type of employment, and educational attainment within the neighbourhood. A total of 2669 couples included in the analysis had all undergone treatment for up to 2.5 years. Live birth rates were not available for all treatments and were not considered as the main outcome. They were, however, used in an additional analysis involving a subset of the data (n = 2312 couples), which consistently showed a significant association between low neighbourhood SEP and lower live birth rate. Couples with low SEP had lower odds of achieving a live birth compared to couples with high SEP (OR = 0.63, 95% CI, 0.48–0.82). Since the start of ART treatment in the Netherlands, three IVF cycles for every patient have been reimbursed by the mandatory health insurance, which makes the accessibility to treatment comparable in a Danish context. The authors explain that financial barriers to IVF access are less likely to explain the observed differences in outcome in the present study (Speksnijder *et al.*, 2024).

Groes *et al.* (2017) showed that there is a large education gradient in ART success in Denmark. Amongst those who access ART services, the highly educated mothers were the most likely to be successful, which is very consistent with our results (Groes *et al.*, 2017). Similarly, Goisis *et al.* (2024) found that Danish mothers with university degrees had a higher chance of achieving a live birth after MAR regardless of parity. Furthermore, families with a higher income were more likely to have a MAR child (Goisis *et al.*, 2024). In the Norwegian register-based study among 1 757 768 live births, of which 32 580 were ART conceived, the results showed that parents who conceived through ART were more likely to be older, married, with the highest levels of income and education (Goisis *et al.*, 2020). Norway is very similar to Denmark regarding the organization of their healthcare system and their access to register data, which makes the study very comparable in a Danish context. Access to ART treatment is generally comparable in the Nordic countries, where treatment is at least partially publicly funded (Capital Region of Denmark, 2020; Ramm *et al.*, 2023).

In the UK study among 3091 women undergoing their first cycle of ART treatment, women from the least deprived areas (Index of Multiple Deprivation; IMD 9–10) had higher risk of achieving a live birth than women from the most deprived areas (IMD 1–2) (aRR= 1.18 [95% CI, 1.00; 1.39]) (Richardson *et al.*, 2020). This indicates that more deprived women are significantly less likely to have a live birth per cycle than less deprived women. Even though the UK differs from Denmark regarding the legislation on fertility treatment, the first cycle of ART treatment is typically NHS-funded but varies according to the postcode lottery (National Health Service (NHS), 2021). Since the study only took the first cycle into account, we assume that the majority of women have received public funding, which strengthens the comparability to our study a bit. However, the study was conducted in a single fertility centre, which may affect the generalizability.

Socioeconomic disparities in live birth after ART treatment are also inconsistent with some studies (Barzilai-Pesach *et al.*, 2006; Mahalingaiah *et al.*, 2011). Two of the above studies have been conducted in the United States, where the same opportunities for receiving ART treatment are not present as in Denmark, which weakened the comparability of our study. In the United States, there is no or very limited public reimbursement of ART treatment, and generally the costs are high (Adamson *et al.*, 2018). The Israeli study did not find social inequality either, which can be explained by Israel’s extensive policy of offering full public funding for ART treatment to all Israeli women until the woman has two children with her current partner (Barzilai-Pesach *et al.*, 2006). Israel’s policy and culture regarding ART treatment significantly differ from those of other countries, including Denmark, which also weakened the comparability of our study. The different results could also be explained by their data collection (face-to-face and telephone interviews and questionnaires), resulting in small populations and a high risk of bias.

In the present study, accessing ART treatment was free of charge up to three ‘fresh’ public IVF or ICSI treatments with hormonal stimulation, and fertility clinics were available to couples and women irrespective of their economic capacity or other sociodemographic characteristics. An exception for publicly reimbursed ART treatments applied to couples who already had one or more children together prior to initiating their first ART treatment. However, the analysis in Table 3 showed socioeconomic disparities even when grouped by parity at baseline. As shown, even in this scenario, higher education, income, and attachment to the labour market were associated with a greater probability of achieving a live birth after initiating ART treatment. Even with reimbursement of treatment costs, medications are only partly reimbursed, and time spent in treatment can further influence the possibility of going to work. This implies both direct and indirect economic costs of treatment. Higher SEP probably improves the likelihood of overcoming these barriers (Vassard *et al.*, 2018). For instance, women with higher education levels may have jobs that offer flexibility or accommodations for treatment-related responsibilities, supporting them to continue ART treatment after unsuccessful attempts. Another aspect to consider is the geographical location of fertility clinics, which are mainly concentrated around larger cities in Denmark (Andersen, 2025). The combination of economic, practical, and emotional demands of undergoing fertility treatments likely affects women and couples differently depending on SEP background. Interestingly, we found a slightly weaker association between SEP indicators and live birth after ART treatment during the latest time period, 2007–2017, where regulatory changes had been made in regard to treatment access. It was also a period with increased focus on less strenuous treatment (e.g. frozen embryo transfer, IUI), perhaps helping patients to stay in treatment longer. Technological developments, such as the change from slow-freeze to vitrification made frozen embryo transfer increasingly utilized over the years.

Many couples discontinue ART treatment without achieving a pregnancy or live birth for reasons other than poor prognosis or the cost of treatment (Smeenk *et al.*, 2004; Brandes *et al.*, 2009; Boivin *et al.*, 2012). The results from Table 4 indicated a tendency for women with higher SEP to be more likely to initiate subsequent ART treatment after unsuccessful treatment, regardless of whether public treatment was an opportunity (second and third) or whether it most likely was private treatment (fourth and fifth). Our results can also be explained by the fact that an increasing number of ART treatments implies a continuous selection into treatment, reflecting resilience to the strain of undergoing treatment and treatment side effects, and potentially reflecting better health (Sejbæk *et al.*, 2015; Vassard *et al.*, 2018).

In Denmark, more than half of the women initiating ART treatment have received other types of MAR (e.g. IUI) prior to ART treatment. Therefore, they may have changed their diet, lost weight, stopped smoking, and adjusted other health behaviours before initiating ART treatment to maximize the chance of conception (Vassard *et al.*, 2018). The few deaths during the follow-up can therefore be explained by a healthy population aged 18–45 years. Hence, death was not considered a major factor influencing these analyses, as would be expected in a young study population.

Danish legislation has been revised, and since the end of 2024, up to six publicly funded ART attempts are offered for the first child (Ministry of the Interior and Health of Denmark, 2024). This new health policy agenda could potentially have a positive impact on increasing the number of children born following the initiation of ART treatment, as up to six ART treatments increase the likelihood of having a live birth (Smith *et al.*, 2015). Furthermore, three ART attempts for women and couples’ second child have been publicly funded by the end of 2024 (Ministry of the Interior and Health of Denmark, The Health Committee, 2024). Whether these measures will increase or decrease social inequality need to be investigated further.

### Strengths and limitations

A key strength of this study is the large, nationwide population of fertility patients, which enabled analyses of subgroups of women undergoing ART. The linkage of individual health data with sociodemographic information from population registers allowed for adjustment for potential confounders. However, the observational design of the study limits the ability to draw conclusions on causality. On the other hand, the study design ensured temporality and made it possible to adjust for potential confounders, which meant that we would approach it. The higher HR of achieving a live birth among women with higher SEP is unlikely to be attributable to delayed conception, as age was accounted for in the analyses. A true association between SEP and successful ART treatment outcome is supported by a clear trend across socioeconomic indicators, with the highest HR of achieving a live birth by women with higher education, income, and attachment to the labour market, even when reducing the size of the data material in the analyses (Table 3). Due to the statistical power of the study, it was also possible to categorize seven education levels, which allowed us to see a clear gradient.

It is well-known that some women become spontaneously pregnant after initiating ART treatment (Pinborg *et al.*, 2009). A Danish study has investigated different diagnostic categories in relation to spontaneous conceptions after initiating ART treatment (Pinborg *et al.*, 2009). The highest delivery rates after spontaneous conceptions were seen in women with anovulatory infertility and unexplained infertility, which were significantly different from those diagnosed with tubal pathology, mixed female infertility, and mixed female and male factor. Furthermore, 10.7% (16/149) of those with a spontaneous conception had changed their partner during the 5-year follow-up period, possibly to a partner with better fertility. There is a need in this field to specify how births are connected to ART. It is a strength that this study involves all live births after initiating ART treatment, also due to the fact that the ultimate goal for couples or women is to achieve a live birth, regardless of whether the pregnancy is treatment-related or not (Daya, 2005).

This is the first study to examine the association between SEP and achieving a live birth following ART using near-complete registration of Danish ART patients up to 2017, as IVF was performed only in very small numbers in Denmark before 1994. During 1994–2017, all ART treatments performed in Danish public and private fertility clinics were recorded. In addition, because a large proportion of first-time ART treatments in Denmark are offered through the public health-care system, our study was able to include women from both lower and higher socioeconomic backgrounds.

Another major strength of this study is the use of time-to-event models to examine the association between SEP and achieving a live birth after initiating ART treatment, which enabled us to take time and competing risks into account. Our study is the first to examine the impact of a variety of socioeconomic factors on the association between SEP and achieving a live birth after initiating ART treatment. However, as seen in Table 2, labour market attachment correlates with education and income. All three socioeconomic variables are indirect measures of health-related behaviour, which may lead to similarity in associated health outcomes (Morris *et al.*, 2011). A concern of close correlation and even causal implications is mostly seen between labour market attachment and income level, as unemployment directly causes a reduction in income level (Stronks *et al.*, 1997). SEP factors were registered at first ART treatment only, which does not account for the complexity and changes that might occur during the study period. However, given the relatively short median follow-up period, we would not expect a systematically differential impact on the results.

It cannot be ruled out that other baseline factors were potential confounders and, therefore, would have been relevant to consider. Factors such as a change in partnership status or health status during the course of ART treatment are relevant causes for terminating treatment, but in this regard are considered mediating factors and thus not considered potential confounders. A limitation is that our study does not include non-ART treatment (e.g. IUI), as the national IVF register only included these treatments from 2007 onwards. As we find socioeconomic disparities in the use of ART treatments (Brautsch *et al.*, 2023) and in live births after ART treatment initiation in the public health care sector, we consider other aspects than direct costs for patients to have an impact on the use of public fertility treatment. Despite less invasive treatment methods needing fewer medical consultations, we speculate whether geographical distance to treatment and difficulties in combining work life and treatment would also have an impact when using less invasive treatment methods.

## Conclusion

In this study, a consistently higher likelihood of achieving a livebirth after ART treatment with higher SEP levels was found across public/private clinics, parity, and marital status, and there was a consistently higher treatment continuation with higher SEP. Free access to fertility treatment in public healthcare should ideally provide equal access. Difficulties encountered during treatment that challenge certain patients more than others based on their socioeconomic background need to be addressed on both a societal and clinical level to ensure equal access to treatment.

There is a need to focus on systematically developing and testing methods to manage and reduce treatment-related burdens, including practical and resource considerations. Because of the emotional consequences of infertility and the stressful nature of ART treatment, there may be a need for healthcare professionals to identify patients with high practical, resource, and emotional load. Support identifying and handling of potential barriers as part of the medical treatment process may reduce social inequality. However, it is likely not only the treatment in the clinic that determines the observed inequality, and there is a need for studies investigating various mechanisms.

## Supplementary Material

hoag032_Supplementary_Data

## Data Availability

The data analyzed in this article cannot be shared publicly as the data sources are national register information established utilizing the unique personal identification number assigned to all citizens in Denmark. Thus, DANAC II Cohort register data were linked at the individual level. The use of microdata from the national registers accessed from Statistics Denmark follows the rules and regulations of the General Data Protection Regulation. Due to data security and legislation, no data are available.
