# Supplementary material for: Socioeconomic disparities in achieving a live birth after initiating ART treatment: a national register-based study among women in Denmark
Source: Hum Reprod Open. 2026 Apr 9;2026(3):hoag032. doi: 10.1093/hropen/hoag032 (PMC13198383; doi:10.1093/hropen/hoag032)
Supplement: hoag032_Supplementary_Data [file hoag032_supplementary_data.docx]

**Supplementary Table S1. Association between socioeconomic position and first livebirth after initiation of ART in Cox regression reported in HR with 95% CI (The DANAC II cohort 2007–2017).**

|  |  |
| --- | --- |
|  | **Model 2 *)**  **2007–2017**  **HR [95% CI]** |
| **Education** |  |
| Primary school | 1 |
| High school | 1.43 [1.34; 1.53] |
| Vocational education | 1.35 [1.28; 1.43] |
| Short higher education | 1.65 [1.53; 1.77] |
| Medium higher education | 1.74 [1.65; 1.84] |
| Long higher education | 2.12 [2.00; 2.25] |
| Research education | 2.20 [1.94; 2.51] |
| **Labour market attachment**)** |  |
| Employed | 1 |
| Outside the workforce | 0.80 [0.75; 0.85] |
| Unemployed | 0.70 [0.66; 0.75] |
| Student | 0.91 [0.86; 0.97] |
| **Income***)** |  |
| 1. Quintile (lowest) | 1 |
| 2. Quintile | 1.11 [1.05; 1.17] |
| 3. Quintile | 1.16 [1.11; 1.23] |
| 4. Quintile | 1.33 [1.26; 1.39] |
| 5. Quintile (highest) | 1.54 [1.46; 1.61] |
| *Multivariate analysis for education, labour market attachment and income adjusted for age and origin  **Labour market attachment was recorded as either employed, unemployed or outside the labour market attachment due to sickness pay, education allowance, leave benefit or early retirement. Students were recorded as a separate group.  ***Income was recorded as an equivalized annual household income used as a relative measure divided into quintiles.  HR, Hazard Ratio. | |
